# Supplementary figures and images for: LH/hCG Regulation of Circular RNA in Mural Granulosa Cells during the Periovulatory Period in Mice
Source: Int J Mol Sci. 2023 Aug 23;24(17):13078. doi: 10.3390/ijms241713078 (PMC10488058; doi:10.3390/ijms241713078)

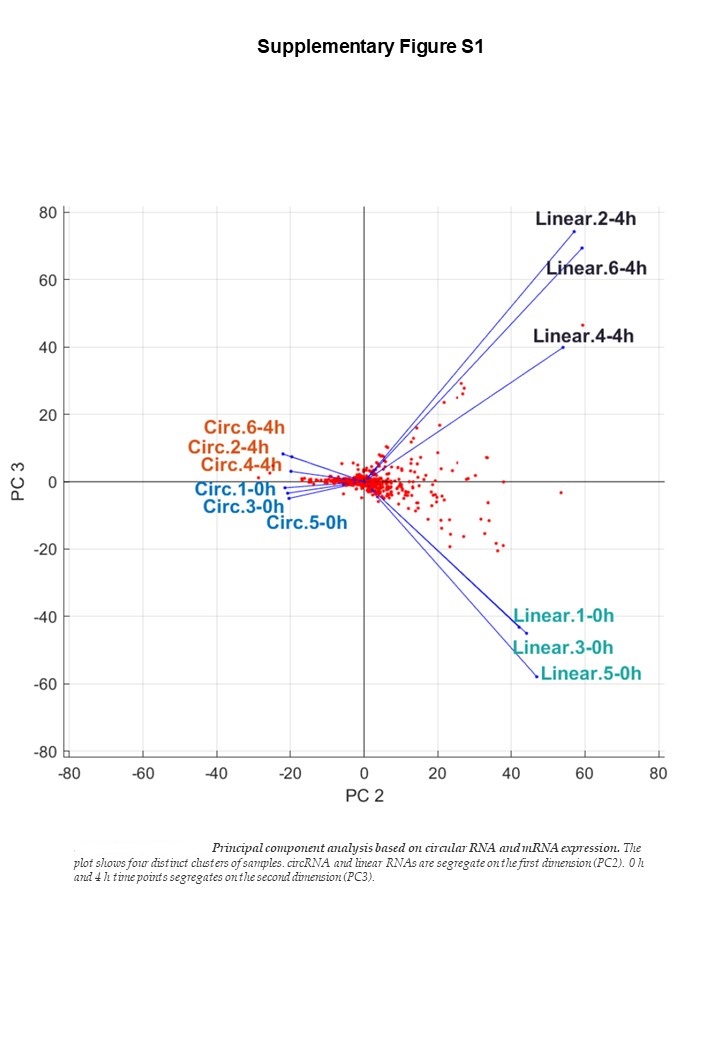

Supplement: Supplementary file 1 [file ijms-24-13078-s001.zip › Supplementary Figure S1.JPG]

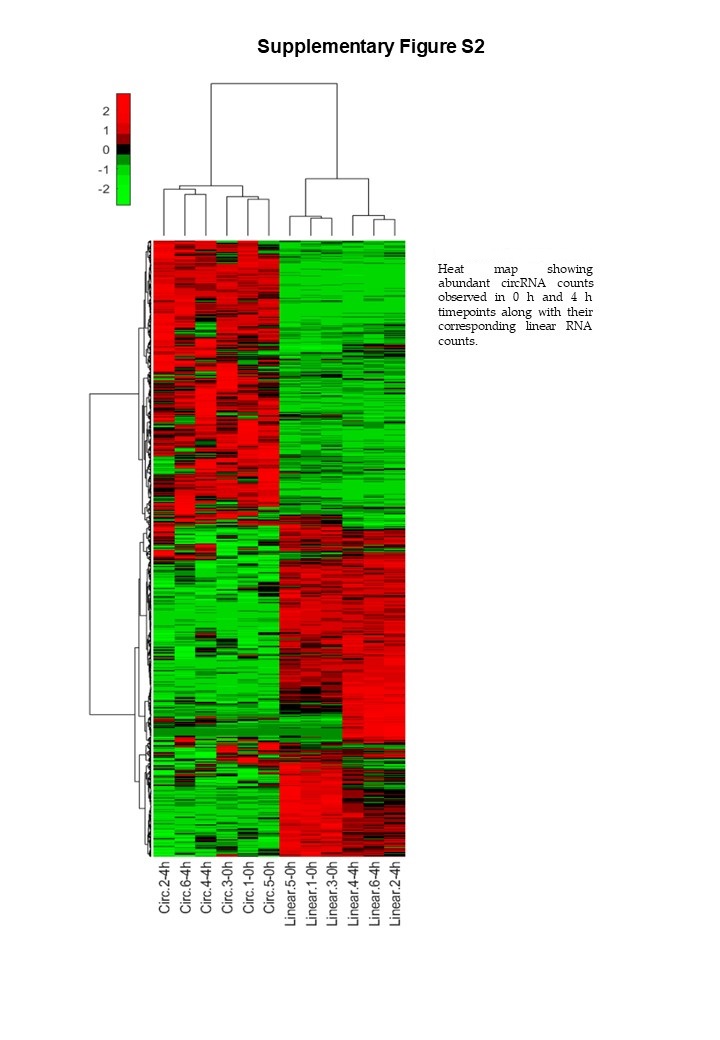

Supplement: Supplementary file 1 [file ijms-24-13078-s001.zip › Supplementary Figure S2.JPG]

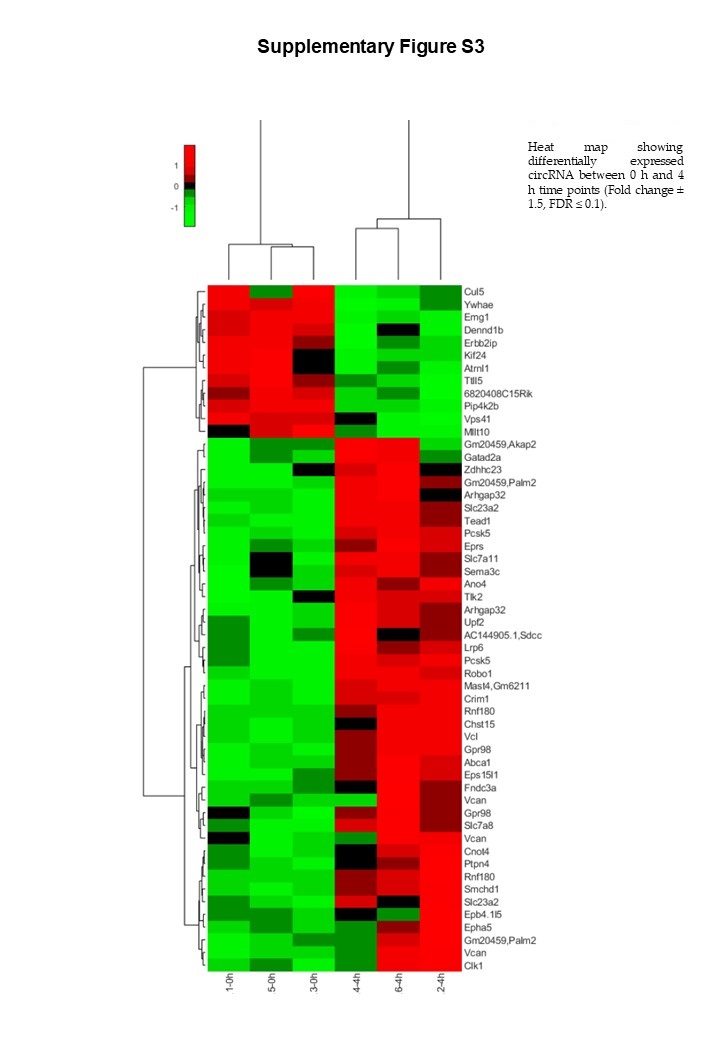

Supplement: Supplementary file 1 [file ijms-24-13078-s001.zip › Supplementary Figure S3.JPG]

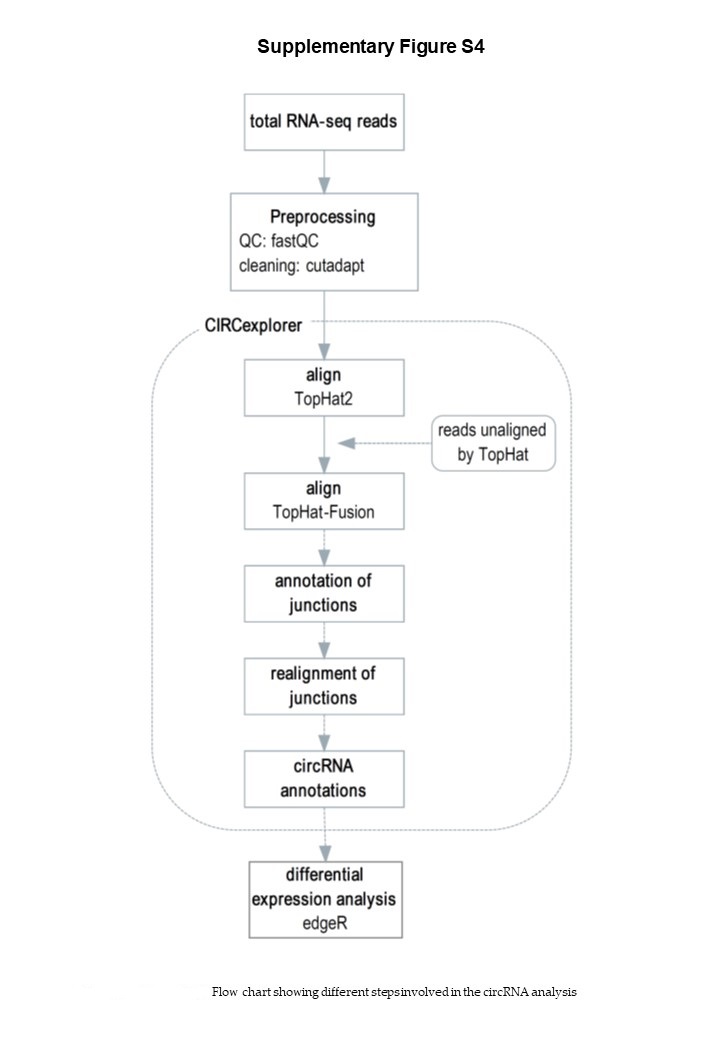

Supplement: Supplementary file 1 [file ijms-24-13078-s001.zip › Supplementary Figure S4.JPG]
